# Factors associated with hospitalizations and deaths of pregnant women from Paraná due to COVID-19: a cross-sectional study

**DOI:** 10.1590/1980-549720240005

**Published:** 2024-02-05

**Authors:** Larissa Silva Bergantini, Sueli Mutsumi Tsukuda Ichisato, Maria Aparecida Salci, Marcela Maria Birolim, Márcia Lorena Alves dos Santos, Carla Franciele Höring, Roberta Rossa, Luiz Augusto Facchini

**Affiliations:** I Universidade Estadual de Maringá, Postgraduate Program in Physiological Sciences – Maringá (PR), Brazil.; II Universidade Estadual de Maringá, Postgraduate Program in Nursing – Maringá (PR), Brazil.; III Centro Universitário Guairacá, Postgraduate Program in Health Promotion – Guarapuava (PR), Brazil.; IV Universidade Estadual de Maringá, Department of Statistics – Maringá (PR), Brazil.; V Universidade Federal de Pelotas, Department of Social Medicine – Pelotas (RS), Brazil.

**Keywords:** COVID-19, Cross-sectional studies, Pregnant women, Hospitalization, Intensive care units, Maternal mortality, COVID-19, Estudos transversais, Gestantes, Hospitalização, Unidades de terapia intensiva, Mortalidade materna

## Abstract

**Objective:**

To analyze the factors associated with hospitalization in the ward and intensive care unit (ICU), and with death from COVID-19 in pregnant women with confirmed cases.

**Methods:**

Observational, cross-sectional study, carried out with data from pregnant women with a confirmed case of COVID-19 from the Influenza Epidemiological Surveillance Information System and the Paraná’s state COVID-19 notification system. The association between the independent and dependent variables (hospitalization in the ward and ICU, and death) was investigated using the Poisson regression model with robust variance.

**Results:**

4,719 pregnant women comprised the study population. 9.6 and 5.1% were hospitalized in wards and ICU, respectively. 1.9% died. There was an association between advanced maternal age and hospitalization in wards (PR=1.36; 95%CI 1.10–1.62) and ICU (PR=2.25; 95%CI 1.78–2.71), and death (PR=3.22; 95%CI 2.30–4.15). An association was found between the third trimester and hospitalization in wards (PR=5.06; 95%CI 2.82–7.30) and ICU (PR=6.03; 95%CI 3.67–8.39) and death (PR=13.56; 95%CI 2.90–24.23). The second trimester was associated with ICU admission (PR=2.67; 95%CI 1.36–3.99). Pregnant women with cardiovascular disease had a higher frequency of hospitalization in wards (PR=2.24; 95%CI 1.43–3.05) and ICU (PR=2.66; 95%CI 1.46–3.87). Obesity was associated with ICU admission (PR=3.79; 95%CI 2.71–4.86) and death (PR=5.62; 95%CI 2.41–8.83).

**Conclusions:**

Advanced maternal age, the end of the gestational period and comorbidities were associated with severe COVID-19.

## RESUMO

**Objetivo:**

Analisar os fatores associados à hospitalização em enfermaria e unidade de terapia intensiva (UTI), e ao óbito pela COVID-19 em gestantes com caso confirmado.

**Métodos:**

Pesquisa observacional, transversal, realizada com dados de gestantes com caso confirmado para COVID-19 provenientes do Sistema de Informação de Vigilância Epidemiológica da Gripe e do Sistema Estadual Notifica COVID-19, do Paraná. Investigou-se a associação entre variáveis independentes e dependentes (hospitalização em enfermaria e UTI, e óbito) pelo modelo de regressão de Poisson com variância robusta.

**Resultados:**

4.719 gestantes compuseram a população do estudo; 9,6 e 5,1% foram hospitalizadas em enfermaria e UTI, respectivamente; 1,9% evoluíram para óbito. Houve associação entre a idade materna avançada e internação em enfermaria (RP=1,36; IC95% 1,10–1,62) e UTI (RP=2,25; IC95% 1,78–2,71), e óbito (RP=3,22; IC95% 2,30–4,15). Verificou-se associação entre o terceiro trimestre gestacional e hospitalização em enfermaria (RP=5,06; IC95% 2,82–7,30) e UTI (RP=6,03; IC95% 3,67–8,39) e óbito (RP=13,56; IC95% 2,90–24,23). O segundo trimestre associou-se à internação em UTI (RP=2,67; IC95% 1,36–3,99). Gestantes com cardiopatia apresentaram maior frequência de hospitalização em enfermaria (RP=2,24; IC95% 1,43–3,05) e UTI (RP=2,66; IC95% 1,46–3,87). A obesidade foi associada à admissão em UTI (RP=3,79; IC95% 2,71–4,86) e ao óbito (RP=5,62; IC95% 2,41–8,83).

**Conclusão:**

A idade materna avançada, o final do período gestacional e comorbidades foram fatores associados a quadros graves de COVID-19.

## INTRODUCTION

In March 2020, the World Health Organization recognized the Coronavirus disease 2019 (COVID-19) pandemic^1^, caused by Severe Acute Respiratory Syndrome Coronavirus 2 (SARS-CoV-2)^2^, as a Public Health Emergency of International Concern. The disease has multisystemic manifestations^3^, and its spectrum varies from asymptomatic, mild and moderate, to severe and fatal stages^4^.

Pregnant and postpartum women, who were initially not considered more prone to severe conditions compared to the general population^5^, constitute a vulnerable group for severe forms of the pathology^6^. Immunological, anatomical, and physiological changes generate a state of greater susceptibility to respiratory infections in women during the gestational period^7,8^. Furthermore, unfavorable prognoses were observed in pregnant women affected by other coronaviruses from the family of the etiological agent of COVID-19^9^.

In relation to women of reproductive age, who were not pregnant, symptomatic pregnant women were at increased risk of developing COVID-19 in its severe form^10^, and, consequently, of admission to an intensive care unit (ICU)^10–13^, of needing mechanical ventilation^10–12^, and of death^10,13^.

In Brazil, more than 24 thousand cases of severe acute respiratory syndrome (SARS) due to COVID-19 have been recorded in pregnant and postpartum women, according to data from the Brazilian Obstetric Observatory SARS (*Observatório Obstétrico Brasileiro SRAG* – OOBr SRAG) updated on August 2^nd^, 2023. 23.7% of these women required intensive care, and 8.4% of them died^14^.

Pregnant women from low- and middle-income countries are more vulnerable to the negative outcomes of COVID-19, such as death^15^. Added to this, the distribution of the number of maternal deaths due to the pathology in Brazilian states appears to be asymmetric, possibly due to socioeconomic differences resulting in unequal access to health services^16,17^. This points to the relevance of conducting research with this population in the Brazilian context.

Considering that pregnant women are more susceptible to the worsening of COVID-19, it is crucial to determine the conditions related to severe cases of the disease. In this context, the presence of comorbidities can act as a risk factor, as well as advanced maternal age^18^, defined by the Brazilian Ministry of Health as the pregnancy of women aged 35 years old or older^19^.

The influence of the gestational period on the predisposition to severe manifestations of COVID-19 is a controversial point in the literature: there are studies that observed associations between the trimester of pregnancy and the severity of the disease^20,21^, and others that did not find this relationship^18,22^.

Furthermore, despite advances in scientific knowledge on this topic, analyses developed in Brazil involving pregnant and postpartum women predominantly investigated the risks of morbidity and mortality in the in-hospital setting, based on data from the Influenza Epidemiological Surveillance Information System (*Sistema de Informação de Vigilância Epidemiológica da Gripe* – SIVEP-Gripe), which monitors SARS cases^17,23^.

This research aimed to contribute to the understanding of the relationship between COVID-19 and pregnancy by exploring the factors associated with severe conditions, considering pregnant women affected at different levels of severity, including non-hospitalized cases. Thus, the objective was to analyze the factors associated with hospitalization in the ward and ICU, and death from COVID-19 in pregnant women with a confirmed case.

## METHODS

### Study design

Observational, cross-sectional research, which used secondary data from two COVID-19 epidemiological surveillance databases: SIVEP-Gripe and the specific information system of the state of Paraná, called *Sistema Estadual Notifica COVID-19* (Notifica COVID-19).

The first 18 months of the pandemic were defined as the investigation period. Specifically, the study comprised pregnant women notified in SIVEP-Gripe between March 29^th^, 2020 (date of the first registration of a pregnant woman from Paraná in this base) and September 29^th^, 2021, and those registered in Notifica COVID-19, from March 30^th^, 2020 (date of the first notification of a pregnant woman from Paraná in the state system) to September 30^th^, 2021. The date of notification of the case in the aforementioned systems was considered for the inclusion of pregnant women in the study.

The research was described based on the guidelines of the Strengthening the Reporting of Observational Studies in Epidemiology (STROBE) initiative^24^.

### Study background

The state of Paraná, in South Brazil, is the most populous in the region, and fifth in the Brazilian rank according to data from the 2022 Demographic Census^25^. The human development index (HDI) of Paraná is equivalent to 0.769, the seventh highest in the country^26^. Until August 2023, the state had the fourth highest number of confirmed cases and deaths from COVID-19 in Brazil^27^.

### Participants

The study population consisted of pregnant women residing in and notified by the state of Paraná, with a confirmed case of COVID-19, aged between 10 and 49 years, and without reservations regarding the gestational trimester. Women who did not require hospitalization due to the disease were included, in addition to those who required hospitalization, in a ward or ICU, regardless of whether the condition progressed to cure or death.

Women with incomplete information on the following were excluded from the analysis: gestational age, date of first symptoms, outcome (cure or death), and place of hospitalization (ward or ICU). Asymptomatic hospitalized pregnant women were not included, with the aim of avoiding hospitalizations caused by obstetric reasons, such as childbirth.

**Figure 1:**
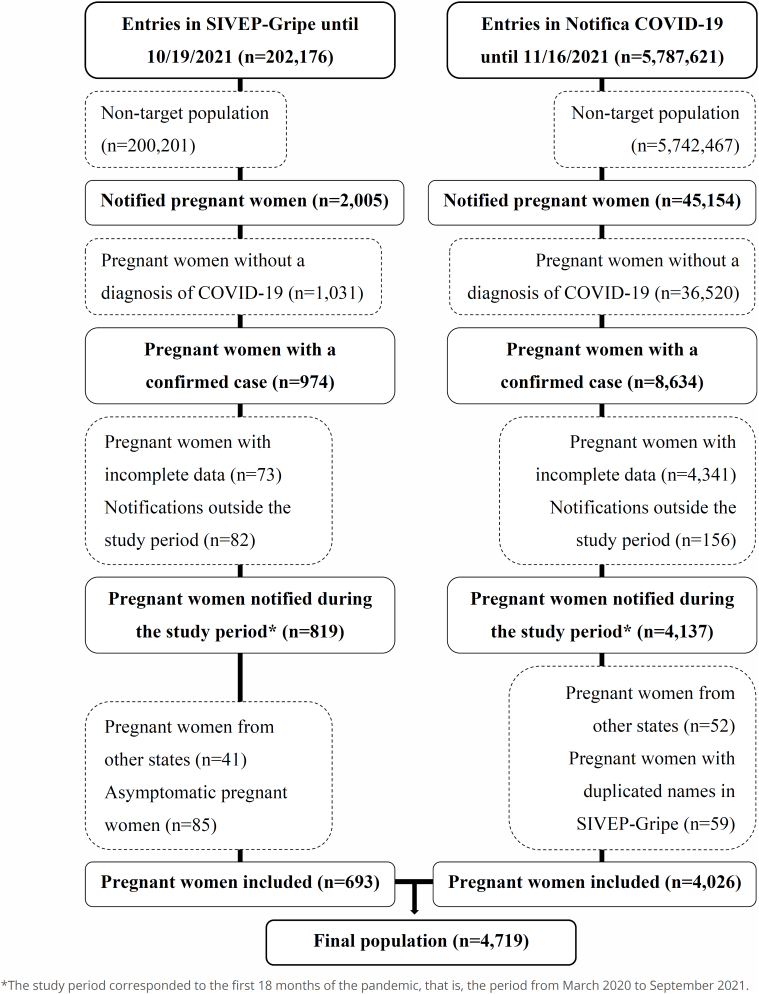
Flowcharts for sample selection of pregnant women from the SIVEP-Gripe (left) and Notifica COVID-19 (right) databases.

The processes for defining the study population are illustrated in Figure 1.

### Data Sources

Information was extracted from SIVEP-Gripe and Notifica COVID-19 databases combined. SIVEP-Gripe is a nationwide information system that monitors SARS cases, whose notification is mandatory in the country. In 2020, COVID-19 surveillance was incorporated into SIVEP-Gripe^28^.

Records of mild and asymptomatic cases of COVID-19 in Brazilian territory were sent to the e-SUS Notifica^28^ system. In the state of Paraná, however, the State Health Secretariat (*Secretaria de Estado da Saúde* – SESA/PR) implemented Notifica COVID-19, its own system for monitoring mild occurrences. Subsequently, data from Notifica COVID-19 is passed on to e-SUS Notifica.

For this research, data were collected from non-hospitalized pregnant women, treated only on an outpatient basis, through the Notifica COVID-19 database. Occurrences of hospitalization in the ward and ICU were identified using the SIVEP-Gripe database. In order to avoid duplicates, the pregnant women’s names were checked in both databases and, in case of duplicity, only the SIVEP-Gripe data were kept.

Versions of databases that are not available to the general public and that contain sensitive data were analyzed. The SIVEP-Gripe database provided to the authors by SESA/PR covered notifications made until October 19^th^, 2021, and the Notifica COVID-19 database until November 16^th^, 2021. The information was collected in December 2021.

### Characteristics

Three outcomes were determined for the analysis: hospitalization in the ward, hospitalization in the ICU, and death due to COVID-19. In the present research, response variables are counts and, therefore, have a discrete quantitative nature. They can be expressed as: Y=number of pregnant women hospitalized in the ward, Z=number of pregnant women hospitalized in the ICU, and W=number of pregnant women who died.

The objective was to model the Y/t=rate of pregnant women hospitalized in the ward; Z/t=rate of pregnant women admitted to the ICU; and W/t=rate of pregnant women who died, where t corresponds to the total number of pregnant women in Paraná diagnosed with COVID-19 in the first 18 months of the pandemic.

To construct the analysis, only the explanatory variables common to both databases were selected, namely: age range, subdivided into younger than 35 years (reference category) and 35 years or more; gestational period, categorized as first (reference category), second, and third trimesters; and the following comorbidities: heart disease, diabetes, obesity and others, all dichotomized as yes or no (reference category). Uncompleted (ignored) records for comorbidities were included in the "no" group.

The age range variable was categorized based on the advanced maternal age stratification proposed by the Ministry of Health^19^. The race/color factor was excluded from the inferential analysis due to the high number of incomplete cases.

### Statistical methods

Data were subjected to descriptive analysis in the Excel^®^ software to calculate median, mean, standard deviation (SD) and absolute and relative frequencies. Inferential statistical analysis was performed using R^®^ software, version 4.1.1.

The Poisson regression model with robust variance was used to investigate possible associations between explanatory variables and responses based on the estimation of crude and adjusted prevalence ratios (PR) and the respective 95% confidence intervals (95%CI).

The variables that presented p-value <0.20 in the crude analysis were maintained in the multiple model. Backward selection was used to determine which variables would remain in the final model, with non-significant variables (p-value>5%) being removed one by one from the final model. The association between variables was considered significant when the 95% confidence interval did not include the value 1.00.

The Wald test was applied to verify the significance of the estimated coefficients. The quality of fit of the models was evaluated according to deviance and the likelihood ratio test (significance level of 5%).

### Ethical aspects

The research was approved with Certificate of Presentation of Ethical Appreciation (*Certificado de Apresentação de Apreciação Ética* – CAAE) number 34787020.0.0000.0104/2020, by protocol number 4.165.272/2020.

## RESULTS

A total of 4,719 pregnant women with COVID-19 made up the study population. Women’s mean age was 27.8 years (SD=6.4; median=27); 25.4% were in the first, 38.1% in the second, and 36.5% in the third trimester of pregnancy; 40.5% were white, but 45.5% were missing this information.

Furthermore, 586 (12.4%) women were asymptomatic at the time of notification, and 4,133 (87.6%) had some symptoms. Most common signs and symptoms included coughing (61.9%), sore throat (41.8%), fever (32.2%), dyspnea (23.1%), symptoms related to the gastrointestinal tract (diarrhea, nausea or vomiting) (11.9%), and oxygen saturation below 95% (7.2%).

Of the total number of infected pregnant women, 4,026 (85.3%) did not require hospitalization and received outpatient care; 693 (14.7%) were hospitalized. Considering all those notified, 453 (9.6%) women were treated in the ward and 240 (5.1%) in the ICU.

The majority of patients (98.1%) recovered from the disease. 92 (1.9%) deaths from COVID-19 were recorded, 87 among those hospitalized (94.6%), 12 in the ward and 75 in the ICU, and five (5.4%) among pregnant women who were not hospitalized, registered in Notifica COVID-19.

Furthermore, 32.5% of those hospitalized for COVID-19 had more than eight years of education, however, there was a predominance (56.1%) of incomplete records for this variable. Almost all (90.9%) of hospitalized pregnant women lived in the urban area, and 41.1% were part of the Eastern health macro-region, which encompasses Curitiba (Paraná’s capital) and its metropolitan region.

The average length of stay was approximately nine days (SD=11.5; median=5), with 42.6% of those hospitalized not needing to use ventilatory support. Among those assisted in the ward, 58.3% did not receive ventilatory therapy, while the majority (85%) of patients in the ICU used the treatment. Considering the 87 hospitalized women who died, eight (9.2%) did not use ventilatory support. These eight patients were hospitalized for an average of nine days, and most were admitted to the hospital in the first half of 2021.

Of the total number of patients who required hospitalization, 108 (15.6%) received invasive ventilation. Among those hospitalized who used invasive ventilatory support, 49 (45.4%) survived and 59 (54.6%) died.

In Table 1, the prevalence of hospitalization in the ward and ICU and the proportions of deaths due to COVID-19 are described according to the categories of explanatory variables in relation to the total number of pregnant women reported.

**Table 1:** Prevalence of hospitalization in wards and intensive care units and proportions of deaths in relation to the total population of pregnant women according to the categories of independent variables. Paraná, Brazil, March 2020 to September 2021

Characteristics		Outcomes						Total notifications (n=4,719)
		Hospitalization in a ward (n=453)		Hospitalization in ICU (n=240)		Death (n=92)		
		n	Prevalence (%)	n	Prevalence (%)	n	Proportion (%)	n
Age range (years)								
	<35	353	9.01	159	4.06	54	1.38	3,918
	≥35	100	12.48	81	10.11	38	4.74	801
Gestational trimester								
	First	42	3.50	17	1.42	3	0.25	1,199
	Second	103	5.72	71	3.94	31	1.72	1,800
	Third	308	17.91	152	8.84	58	3.37	1,720
Comorbidity								
Heart disease								
	No	436	9.36	221	4.75	85	1.83	4,656
	Yes	17	26.98	19	30.16	7	11.11	63
Diabetes								
	No	427	9.35	218	4.77	82	1.80	4,568
	Yes	26	17.22	22	14.57	10	6.62	151
Obesity								
	No	429	9.42	202	4.44	74	1.62	4,554
	Yes	24	14.55	38	23.03	18	10.91	165
Others								
	No	443	9.64	231	5.03	87	1.89	4,597
	Yes	10	8.20	9	7.38	5	4.10	122

ICU: intensive care unit.

Table 2 presents the crude prevalence ratios of the survey response variables according to the explanatory variables. In Table 3, the adjusted prevalence ratios are shown according to the variables that remained in the multiple model for each outcome.

**Table 2:** Crude prevalence ratios of outcomes according to age group, gestational trimester, and comorbidities. Paraná, Brazil, March 2020 to September 2021.

Characteristics		Outcomes					
		Hospitalization in a ward		Hospitalization in ICU		Death	
		PRc (95%CI)	p*	PRc (95%CI)	p*	PRc (95%CI)	p*
Age range (years)							
	<35 (reference)	1.00	–	1.00	–	1.00	–
	≥35	1.39 (1.10–1.72)	0.003	2.49 (1.90–3.24)	<0.001	3.44 (2.26–5.19)	<0.001
Gestational trimester							
	First (reference)	1.00	–	1.00	–	1.00	–
	Second	1.63 (1.15–2.36)	0.007	2.78 (1.68–4.88)	<0.001	6.88 (2.46–28.68)	<0.001
	Third	5.11 (3.75–7.16)	<0.001	6.23 (3.89–10.68)	<0.001	13.48 (4.99–55.24)	<0.001
Comorbidity							
Heart disease							
	No (reference)	1.00	–	1.00	–	1.00	–
	Yes	2.88 (1.70–4.52)	<0.001	6.35 (3.85–9.87)	<0.001	6.09 (2.56–12.22)	<0.001
Diabetes							
	No (reference)	1.00	–	1.00	–	1.00	–
	Yes	1.84(1.21–2.68)	0.002	3.05 (1.91–4.62)	<0.001	3.69 (1.79–6.77)	<0.001
Obesity							
	No (reference)	1.00	–	1.00	–	1.00	–
	Yes	1.54(1.00–2.28)	0.038	5.19(3.62–7.25)	<0.001	6.71(3.89–10.97)	<0.001
Others							
	No (reference)	1.00	–	1.00	–	1.00	–
	Yes	0.85(0.42–1.50)	0.612	1.47 (0.70–2.69)	0.258	2.17(0.76–4.81)	0.092

* Regarding the Wald Test; ICU: intensive care unit; PRc: Crude prevalence ratio; 95%CI: 95% confidence interval.

**Table 3:** Adjusted prevalence ratios of outcomes according to the variables that remained in the final model. Paraná, Brazil, March 2020 to September 2021.

Characteristics		Outcomes					
		Hospitalization in a ward		Hospitalization in ICU		Death	
		PRa* (95%CI)	p†	PRa* (95%CI)	p†	PRa*(95%CI)	p†
Age range (years)							
	<35 (reference)	1.00	–	1.00	–	1.00	–
	≥35	1.36(1.10–1.62)	<0.001	2.25(1.78–2.71)	<0.001	3.22(2.30–4.15)	<0.001
Gestational trimester							
	First (reference)	1.00	–	1.00	–	1.00	–
	Second	1.63(0.92–2.34)	0.028	2.67(1.36–3.99)	<0.001	6.74(0.88–12.61)	<0.001
	Third	5.06(2.82–7.30)	<0.001	6.03(3.67–8.39)	<0.001	13.56(2.90–24.23)	<0.001
Comorbidity							
Heart disease							
	No (reference)	1.00	–	1.00	–	–	–
	Yes	2.24(1.43–3.05)	<0.001	2.66(1.46–3.87)	<0.001	–	–
Obesity							
	No (reference)	–	–	1.00	–	1.00	–
	Yes	–	–	3.79(2.71–4.86)	<0.001	5.62(2.41–8.83)	<0.001

* All variables were adjusted for those that remained in the multiple model;

† Regarding the Wald Test;

ICU: intensive care unit; PRa: Adjusted prevalence ratio; 95% CI: 95% confidence interval.

There was no association between diabetes, other comorbidities and the three response variables. Obesity did not remain in the final model for the response variable hospitalization in the ward, and heart disease did not remain in the death outcome, as both presented p-value>5% (backward selection).

The deviance test applied to evaluate the quality of fit of the models obtained p-values>5%, demonstrating the suitability of the proposed models for the three outcomes.

The adjusted analysis identified that pregnant women aged 35 or over were almost 1.4 times more likely to be hospitalized in a ward (95%CI 1.10–1.62) compared to patients below this age range.

Pregnant women in the third trimester were approximately five (95%CI 2.82–7.30) times more likely to be admitted to the ward compared to those in the first trimester. There was no statistically significant difference between women in the second and first trimesters for this outcome. Furthermore, having heart disease doubled (95%CI 1.43–3.05) the probability of a pregnant woman with COVID-19 requiring hospitalization in a ward.

The probability of admission to the ICU was increased by approximately two (95%CI 1.78–2.71) times among pregnant women of advanced maternal age. Compared to women in the first trimester, the probability of admission to the ICU for those in the second and third trimesters increased by almost three and six times, respectively.

Patients with obesity were more likely to be hospitalized in the ICU, with a probability practically four (95%CI 2.71–4.86) times higher than those without the comorbidity. Having heart disease increased the probability of needing intensive care by around two times (95%CI 1.46–3.87).

The probability of death was three (95%CI 2.30–4.15) times higher among pregnant women aged 35 years old or older. Being in the third trimester increased the chance of death from COVID-19 by approximately 13 (95%CI 2.90–24.23) times, however, there was no statistically significant difference with regard to the second and first trimesters of pregnancy. The adjusted analysis found an association between obesity and death, increasing the probability of the outcome by approximately five (95%CI 2.41–8.83) times.

## DISCUSSION

Adjusted analysis showed an association between age equal to or over 35 years old, as well as the third trimester of pregnancy and the three severity outcomes: hospitalization in the ward and ICU, and death due to COVID-19. The second trimester was related to the need for intensive care. Having a heart disease increased the probability of admission to the ward and ICU. There was an association between obesity and ICU hospitalization and death.

The use of secondary data implies limitations inherent to this set of information: there was a high number of records with important elements not filled out, which resulted in purification of the study population and made it impossible to assess the impact of race/color on severe outcomes of COVID-19. Ethnic-racial disparities in relation to the risk of infection and mortality have been reported among pregnant women^10^, therefore, the non-inclusion of health determinants in this analysis is a limitation. The probable underreporting of asymptomatic cases is a limiting factor. This research was carried out in a limited context, the state of Paraná, therefore, generalizing the results to other locations with different socioeconomic and health characteristics must be cautious.

A study carried out with 240 pregnant women with confirmed cases of SARS-CoV-2 infection found that hospitalized patients were more likely to have at least one comorbidity, such as hypertension, type two diabetes, obesity, among others^29^.

Similarly, advanced maternal age and the existence of any comorbidity, including obesity, chronic lung disease, and hypertension, stood out as risk factors for the moderate to severe and critical condition of COVID-19 in a cohort consisted of 7,950 pregnant women^22^, in agreement with the present findings. A dose-response effect was found with regard to comorbidities and severe forms of the disease^22^.

Two studies, which specifically evaluated ICU admission as a response variable, observed that advanced age and comorbidities increased the risk of the outcome^23,30^. One of these analyses carried out in Brazil demonstrated that obesity and diabetes were prominent in relation to the risk of ICU admission among comorbidities^23^.

Regarding death from COVID-19, the two studies cited^23,30^ corroborated the results obtained here: advanced maternal age and the presence of comorbidities were related to mortality from the disease. A second Brazilian survey reinforced that having associated clinical conditions, such as obesity, diabetes, and cardiovascular disease, increased the risk of death^17^.

Regarding the influence of the gestational period on the prognosis of COVID-19, a systematic review, which summarized the products of 77 studies, did not observe a correlation between advanced gestational age and complications arising from the disease^18^.

Another longitudinal analysis found that the gestational trimester was not associated with an increased risk of developing moderate to severe and critical forms of the pathology. The authors themselves, however, highlighted that the low representation of pregnant women in the first trimester of pregnancy could restrict the understanding of the interference of this variable^22^.

On the other hand, a study carried out in Brazil found a predominance of deaths due to COVID-19 in the third trimester of pregnancy or in the postpartum period^16^. Likewise, the majority of patients hospitalized with the pathology in an analysis carried out in the United States were in the third trimester^29^.

A cohort study developed by North American researchers found that pregnant women affected by COVID-19 were three times more likely to be admitted to the ICU compared to those without the disease, and that the risk was increased by infection during the third trimester^31^. Two review studies also indicated that the last gestational trimester was associated with the severity of the pathology^20,21^. These results converge with those of this research.

The anatomic-functional changes observed mainly in the third trimester, such as those in the respiratory system (increased oxygen demand and limitation of lung expansion, for example)^32,33^, could form the pathophysiological mechanism that would explain the increased susceptibility of pregnant women affected at the end of their pregnancy^34^.

Furthermore, the assertion that pregnancy corresponds to a state of immunosuppression *per se* is inaccurate^34^. In the third trimester of pregnancy until delivery, a pro-inflammatory disposition prevails in the woman’s body. In this sense, it is assumed that infection by SARS-CoV-2 could worsen this process during the final phase of the gestational period^33^.

The most frequent symptoms among pregnant women with COVID-19 investigated in this research were coughing, sore throat, fever, and dyspnea, similar to those identified by other authors^23,35^.

As previously mentioned, in developing countries, the impact of COVID-19 on maternal health may be discrepant compared to developed nations^36^. In Brazil, the significant consequences of the disease in pregnant and postpartum women can be explained, primarily, by clinical issues. In the national territory, the main cause of maternal death is hypertensive disorders^19^, which, combined with COVID-19, can deteriorate the prognosis during the gestational period. Added to this are the high prevalence of overweight and obesity among Brazilian pregnant women, both pro-inflammatory states^17,37^.

Furthermore, the so-called indirect effects of the pandemic on maternal health should also be mentioned^38^. Measures implemented around the world to prevent, control, and treat COVID-19 have resulted in difficulties in transportation and access to healthcare, discontinuity of maternal care services and shortages of human and material resources, such as personal protective equipment and medicines^39^.

Responses to the pandemic, particularly in developing countries, may have intensified pre-existing social disparities and exacerbated chronic deficits in health systems^37,39^. Thus, in Brazil, it is suggested that the significant impact of COVID-19 on maternal health is also linked to precarious obstetric care, access impairment, and social risk^17^.

This research found that around 13% of pregnant women hospitalized in the ICU did not receive ventilatory support; 9.2% of those hospitalized who died did not use the therapy, with the majority of these women being admitted to the hospital in the first half of 2021, between March and June, a period in which there was a significant increase in confirmed cases and deaths due to COVID-19 and, consequently, overload of the Brazilian health system^40^. Five patients who did not survive the disease did not receive hospital care. This highlights that obstacles to accessing intensive care can contribute to the mortality of pregnant and postpartum women^17^.

This study used secondary data from epidemiological surveillance databases for an entire state with a significant sample of women. It is considered that the large representation of patients in the three trimesters of pregnancy contributed to the understanding that pregnant women at the end of the gestational period may be more likely to develop severe cases of COVID-19. Furthermore, this analysis emphasized that pregnant women of advanced maternal age, and equally patients with comorbidities, should receive special attention in the pandemic context.

Identifying vulnerable women by the team involved in maternal and child care enables to develop intervention strategies that can prevent or alleviate injuries resulting from COVID-19. Finally, these findings can contribute to the construction of approaches to the continuing education of health professionals, policy formulation, organization of services, tracking of pregnant women, in addition to contributing to the development of future studies.
